# Overview Study of Partially Observable Hidden Markov Models for Ambient Movement Guidance Support

**DOI:** 10.1155/ijta/8095704

**Published:** 2025-03-04

**Authors:** Shahram Payandeh

**Affiliations:** Networked Robotics and Sensing Laboratory, School of Engineering Science, Simon Fraser University, Burnaby, British Columbia, Canada

**Keywords:** ambient intelligence, decision-making, human–machine interaction, movement guidance, partially observable hidden Markov models (POHMMs)

## Abstract

The study of ambient movement guidance encompasses a multidisciplinary approach to facilitating and guiding individuals, particularly older adults, within their living environments. This involves integration of ambient sensors, such as motion detectors, cameras, or IoT devices, to monitor the movements and activities of individuals in real time. By leveraging these sensors, the system can predict and anticipate the expected movements of the person, allowing for proactive ambient guidance and support. In addition to ambient guidance, robots can also play a role in leading individuals by interfacing through audio prompts or visual cues through their daily activities. However, despite advancements in sensor technology and robotic assistance, uncertainties persist in the monitoring and prediction of movements. These uncertainties can arise from various sources, including sensor noise, occlusions, environmental changes, and inherent variability in human behavior. Addressing these uncertainties requires probabilistic modeling techniques based on partially observable hidden Markov models (POHMMs) and various of its extensions such as POMDP, to effectively capture the dynamic nature of movement patterns and incorporate uncertainty into the decision-making process. This paper presents a detailed overview study of probabilistic framework and how its various interpretation can be used in developing an ambient movement guiding system for supporting individuals, particularly older, in support of ageing-in-place paradigms.

## 1. Introduction

The emerging fields of ambient intelligence and persuasive technology are merging information technology with cognitive science, creating smart environments that can intelligently respond to human actions and even influence behavior. These advancements challenge traditional frameworks that define the relationships and roles between humans and technological artifacts. Reference [1] is one of the earliest work that explores the potential benefits and risks of these technologies and proposes new concepts of agency, freedom, and responsibility. These new concepts are aimed at enhancing our understanding and evaluation of the social roles of ambient intelligence and persuasive technology. The main argument is that these technologies require us to reconsider and blur the boundaries between humans and technology at both conceptual and moral levels. For example, Reference [2] presented solutions for particular problems commuters encounter during the walking phase of their journey in public transport facilities. Commuters get obstructed or have collisions at bottleneck situations, making their walking inefficient and stressful. The aim is to provide enhanced feedback to change commuters' behavior to improve the flow and quality of walking. A set of novel analog and digital design interventions has been developed for various bottleneck situations. In response to a rapidly ageing population, there has been a significant increase in assisted living technologies aimed at enabling safe and independent ageing presented by [3]. Factors such as the growing elderly demographic, rising healthcare costs, caregiver burden, and the value individuals place on independence drive this innovation. This survey reviews the development of ambient-assisted living (AAL) tools for older adults, which are based on the ambient intelligence paradigm. It summarizes the latest AAL technologies, tools, and techniques and discusses current and future challenges in the field. Reference [4] presents a survey that explores the application of ambient technology in healthcare, detailing the necessary infrastructure and technologies, such as smart environments and wearable medical devices. It reviews state-of-the-art AI methodologies used in ambient technology systems, including learning, reasoning, and planning techniques, and discusses how such ambient technology intelligence can assist individuals with physical or mental disabilities or chronic diseases. The survey also highlights successful case studies and examines current and future challenges to guide future research. Reference [5] explores designing ambient playful interactions in public spaces using interactive technology, focusing on encouraging playful behavior in teenagers through 10 interactive installations. From these design cases, three key design values are derived: attracting and engaging passers-by to become players, fostering emergent play to allow for personalized and evolving experiences, and resonating with users' values, emotions, and activities. The article also discusses insights from the cases and provides recommendations for implementing playful interactions in public spaces. Reference [6] addresses the challenges in the visual pervasive ambient information displays due to the limited number of available displays in the environment. They proposed a wearable projector system that constantly projects information displays in front of the user, providing access to both public and personal information on the floor. Routine physical activity promotes healthy ageing but is uncommon among older adults. This research study [7] explored older workers' perceptions of persuasive principles from the persuasive system design (PSD) model integrated into an app designed to encourage physical activity. The study highlights the importance of personalization, credible content, and a sense of similarity in designing effective persuasive apps for older workers [8]. In AAL scenarios, it is crucial to provide support that enhances the quality of life for the elderly, especially through systems that adapt to user behavior changes. Reference [8] introduces a platform with an end-user development environment, enabling older adults and their caregivers to customize Web applications for context-dependent behavior and promote healthy habits. The platform uses sensors to gather information, identify behavior changes, and adapt the user interface and application accordingly. Additionally, it allows for personalization by modifying context-dependent trigger-action rules to better suit individual needs. Reference [9] introduced a novel persuasive system designed to assist individuals in walking with crutches. Using a miniprojector, the system projects digital visual cues onto the ground, enhancing timing without constant manual assistance from therapists. This portable, lightweight solution is aimed at boosting users' self-confidence and motivation in learning to walk with crutches.

The realm of ambient movement guidance support as it can be seen in the above review is slowly seeing advancements in recent years, accelerated by advancement of computational intelligence and sensor technologies. In this context and in various implementations related to modeling and monitoring, aspects based on the general notion of partially observable hidden Markov models (POHMMs) have emerged as a possible framework for modeling and predicting human movements in its environments. This paper presents a study aimed at presenting an overview of the principles and applications of POHMMs for ambient movement guidance support system. Understanding human movements in dynamic environments is pivotal for various applications, ranging from smart navigation systems to healthcare monitoring. Traditional Markov models have been instrumental in modeling sequential data; however, their efficacy in scenarios with partial observability is limited. POHMMs address this limitation by incorporating hidden states, enabling more robust modeling of complex systems where not all variables are directly observable. This inherent capability makes POHMMs particularly suitable for applications requiring real-time decision-making in dynamic and uncertain environments. In this paper, an overview presentation of the theoretical underpinnings of POHMMs elucidating their mathematical formulation and inference algorithms is described. The paper also presents walk-through examples which explores the effect that some of the parameters can have in the overall interpretation, modeling, and performance of the framework.

Previously, there have been some investigations which address various problems which can be associated with the notion of design and developing of ambient guiding support system for support of people movements and activities. For example, the study of the importance of safe and efficient interactions between humans and autonomous systems, emphasizing the need for considering the state of the human in decision-making processes, was presented in [10]. The study proposes an enhancement to the autonomous systems by incorporating the human state into decision-making loops. The development includes frameworks and computational tools for human-in-the-loop systems, employing a probabilistic approach with partially observable Markov decision processes (POMDPs). The overview of the growing interest in monitoring the movements of older adults and detecting anomalies using nonwearable sensing technologies was presented in [11]. The method employed involves utilizing Markov chains (MCs) as a framework for monitoring and estimating sequences of events related to movements, contributing to anomaly detection in sensor networks. The paper addresses the challenge by introducing hidden Markov models (HMMs) for situations where the state cannot be directly measured. A decision theoretic model for interactions between users and cognitive assistive technologies, focusing on tasks important for the elderly, was presented by [12]. The model, a POMDP, collaborates with users to complete activities, monitoring user health and adapting to changes. The POMDP model excels in handling uncertainty, ease of specification, broad applicability, and adaptability to evolving tasks and situations. The paper outlines the model, presents a learning method for training with partially labeled data, and demonstrates its application. The significance of activity recognition in intelligent environments to support individuals in their daily activities particularly focusing on the application of POMDP models was presented in [13]. The proposed method initiates with a psychologically justified description of the task and its environment, derived from empirical data. This description is then integrated with specifications of available sensors to construct a functional prompting system. The paper illustrates the method by applying it to the creation of a system guiding individuals through the process of making a cup of tea in a real-world kitchen. Applications of formalized models and analysis techniques in the development of systems for AAL are shown in [14]. Various modeling tools, including fault trees (FTs), evidential reasoning (ER), evidential ontology networks (EONs), temporal logic, HMMs, and partially observable Markov models (POMDP), are reviewed for their potential in the AAL context. The authors note that different models address different problems and that the choice of a model depends on the formulation of the specific problem. The discussed FTs are recognized for their simplicity, mathematical foundation, and ability to handle uncertainty. ER and EONs offer greater expressiveness but lack the ability to reason about time. Temporal logic efficiently handles temporal aspects but struggles with uncertain or missing data. The two Markov model-based techniques, HMM and POMDP, are noted for their capability to handle uncertainty and time relations. The feasibility, acceptability, and clinical effectiveness of socially assistive robots in supporting people with dementia are assessed in [15]. A systematic review of four categories of robots, namely, companion robots, telepresence communication robots, home care assistive robots, and multifunctional robots, are presented. The conclusion emphasized that while socially assistive robots were generally well received, there was insufficient evidence to demonstrate their clear benefits for individuals with dementia. However, Reference [16] presented a study aimed at investigating the effectiveness of a social robot intervention in improving cognitive function, depression, loneliness, and quality of life among older adults living alone. Results indicated a statistically significant improvement in cognitive function, depression reduction, and decreased loneliness in both the experimental and control groups.

## 2. A General Description of POHMMs

A Markov process, often referred to as MCs, characterizes the dynamic evolution of a system composed, for example, of discrete states. These states, representing aspects like a person's position in a monitoring area or a robot guiding a person, are defined within a controlled grid, reflecting a physical living space. Over time or sampled events, the system transitions between these states, and at each instance, it faces various options for the next state it can occupy. The decision-making process at each instant relies on historical data, serving as prior knowledge or trained models for the system. The choices at each state are encapsulated by a discrete probability distribution over potential transitions to the next state, collectively capturing all possible changes in state [11, 17].

In the conventional definition of the *Markov decision process (MDP)* [18], it is assumed that to leverage prior information for transitioning between states (i.e., a person moving from one position to the next), one must observe or measure the system's occupancy in its current state. However, in many cases, accurately determining (measuring or observing) the system's status and presence in the current state may be impractical through observation alone (i.e., cluttered living spaces or lack sensor placement of sensors due to privacy-related concerns). In such instances, an additional prior model for observation can be created (or generated), introducing some uncertainty regarding the system's presence in the current state. This uncertainty is represented as a prior probability distribution derived from training data (or generative data), characterizing the observation of the system's state at the current moment. As the state is not directly observable, it is termed a hidden or partially observable state, exemplified by estimating a person's position based on the presence or absence within an area rather than exact coordinates.

Using HMMs [19] and their extensions for guiding people in their living space involves leveraging these probabilistic models to make decisions and provide action in terms of guidance based on the analysis of environmental components. HMMs are mathematical models used to describe systems that evolve over time and are observed indirectly through a sequence of observable states. In the context of guiding people in their living space, the living space and the person's movements can be modeled as a sequence of hidden states and observable events. The transitions between hidden states represent the dynamics of the person, environment, intentions, including factors such as room setting, obstacles, or specific locations within the living space. Observable events are the information available to the monitoring model, like sensor readings or environmental cues. These events are influenced by the current hidden state, providing a connection between the hidden and observable layers.

These prior models are expressed in terms of probability distributions, outlining the *state transitions* (i.e., person moving from one position to the next) and the distribution of states given the observation or measurement model. Additionally, a sequence of decisions or actions made by the monitoring system in transitioning between states can be collected, associating utility, such as rewards or pay-offs, with each action. Despite the unobservable nature of the state, this evolving sequence of actions and corresponding observations can be employed in the decision-making process. The stored sequence becomes part of the agent's decision-making prior knowledge, recursively forming *a belief distribution* and, consequently, *a belief value* at the current state and instant. The solution to the MDP is known as a *policy*, defining the best action for each state. Various scenarios, such as limited lifetime, finite horizon, and a one-step decision-making process, can be considered, reflecting the decision-making characteristics. The distribution of observations, including measurements, informs the state transition of the system from the initial state, contributing to the overall decision-making framework which is also referred to as POMDP [20].

Extending HMMs to POMDPs allows for decision-making under uncertainty. In the living space, uncertainty can arise from incomplete information about the environment or the person's intentions. Utilizing sensors like cameras, motion detectors, or IoT devices can provide valuable information about the environment. These sensors can be part of the observation model, enhancing the model's ability to understand the current state. Including context-specific data such as time of day, user preferences, or historical patterns helps refine the model's predictions and recommendations. Extensions may involve adapting the model over time based on learning from user behavior. This adaptability ensures that the guidance system becomes more personalized and accurate as it interacts with the user and the environment. The model can guide individuals through their living space, considering factors like optimal paths, avoiding obstacles, and adapting to changes in the environment. By analyzing historical data, the model can predict user behavior, such as preferred routes, common destinations, or times of high activity [21]. For individuals with specific needs, such as the elderly or those with movement/activity decision impairment challenges, the system can adapt ambient guidance strategies to provide tailored support. Employing HMMs and their extensions for guiding people in their living space involves creating a dynamic, adaptive system that integrates environmental components, user behavior, and probabilistic modeling to offer effective and personalized guidance.

## 3. A Formal Description

The following section highlights some of the formal terminologies which have been defined in the literature in modeling POHMM [20, 22–24] [25]. Let transition function *T* define a mapping from the current state *s* with the associated action *a* to the next state *s*′. For every *s* ∈ *S* and *s*′ ∈ *S* and for every action *a* ∈ *A*, the mapping *T*(*s*, *a*, *s*′), with values between 0 and 1, captures the likelihood that taken action *a* at *s* results in transition state *s*′. For example, the action can be environmental guidance cues created by ambient media or action of the leading robot for when the person is at state *s*, to results in the movement of the person to state *s*′. It is also expressed as probability distribution *T*(*s*′|*s*, *a*) which, for example, it captures the conditional probability distribution of dynamics of the person being at state *s*′ and specifies how the action influences the evolution of the system from *s* over time. This also implies that ∀(*s*, *a*), ∑_*s*′∈*S*_ we have *T*(*s*, *a*, *s*′) = 1. This corresponds to the sum of the probabilities of propagating from the current state *s* to all existent next states *s*′, which are available through the transition *T*, must add-up to be one. In general, we can define a probability distribution for the initial state. Specifically, for each *s* ∈ *S*, *b*(*s*) is the probability at the initial state *s*, in which the person begins such that ∀*s*, ∑_*s*∈*S*_*b*(*s*) = 1; that is, *b*(*s*) is a proper probability distribution over initial states (or *initial belief* distribution).

Similar to the state transition function, one can also define a reward function *R*. For every *s* ∈ *S* and *s*′ ∈ *S* and for every action *a* ∈ *A*, the mapping *R*(*s*, *a*, *s*′) is the expected reward for the action *a* from state *s* to the resulting state *s*′. If we define a goal state *g* which satisfies certain objective when the person is occupying it, we can assign the highest and lowest rewards (e.g., *R*(*s*, *a*, (*s*′ = *g*)) = +1 and *R*(*s*, *a*, (*s*′ = ∅) = 0)) (or a small negative reward) or all other combinations. The reward function can be modified by introducing the concept of discounting through a constant *γ* such that 0 ≤ *γ* ≤ 1. That is, reward in the distant future is down-weighted relative to rewards received in the near future. For example, one can adaptively modify a reward *r* received *t* time steps in the future by *γ*^*t*^*r*.

The observation model (function) *O* describes the probability distribution over observations given the current state and action taken. It is expressed as *𝒪* = (*o*|*s*′, *a*) which accounts for the partial observability of the environment and specifies how the agent's actions affect the information it receives about the state of the environment. For every resulting state *s*′ ∈ *S*, and every action *a* ∈ *A*, and every observation *o* ∈ *𝒪*, *O*(*a*, *s*′, *o*) is the probability that the person will be observed through *o*, by taking action *a* to transition to state *s*′ which can result in a proper probability distribution, that is, ∑_*o*∈*𝒪*_*O*(*a*, *s*′, *o*) = 1.

### 3.1. Belief State

The belief state describes an approach where the decision-maker can encapsulate knowledge about the current state of the person from interacting with the environment in order to form a probability distribution over all possible states [26, 27]. In a typical formulation, updating the belief in the state involves integrating new information obtained from observations up to the current time with the existing belief state in order to form a probability distribution.

The belief state is typically initialized based on a prior knowledge or assumptions about the system (i.e., the person) *b*(*s*). This initial belief distribution represents the uncertainty about the true state of the system before any observations are made. Given the initial belief state *b*(*s*) where *s* represents state at instance *t* and an observation *o* is received, the belief update process involves calculating a new belief state *b*′(*s*′) that incorporates this observation. This update can be accomplished using Bayes' rule [28]. For example, given two disjoint events *A* (state) and *B* (observation), we can write *Pr*(*A*|*B*) = *Pr*(*B*|*A*)*Pr*(*A*)/*Pr*(*B*) where *Pr*(*A*|*B*) would be the posterior probability of the state given the evidence (observation), *Pr*(*B*|*A*) is the likelihood of observing (measuring) the state, *Pr*(*A*) is the prior probability of the state, and *Pr*(*B*) is the probability of observing the evidence (also known as the evidence or marginal likelihood) [28]. The prior belief state *b*(*s*) represents the belief about the state of the system before observing any evidence. The likelihood *Pr*(*o*|*s*) represents the probability of observing evidence *o* given the true state *s*. The transition model *Pr*(*s*′|*s*, *a*) represents transitioning from state *s* to *s*′ given the action *a*.

Applying Bayes' rule [28], the posterior belief state *b*′(*s*′) after observing evidence *o* can be computed as

(1) $$\begin{array}{c} b^{\prime }\left(s^{\prime }\right)=\frac{Pr\left(O\left|s^{\prime }\right.\right)\cdot \sum_{S}Pr\left(s^{\prime }\left|s,a\right.\right)\cdot b\left(s\right)}{Pr\left(o\right)}. \end{array}$$

The term ∑_*S*_*Pr*(*s*′|*s*, *a*) · *b*(*s*) represents the marginalization [29] of the transition probability and the prior belief state. For each possible state *s*, we compute the probability of transitioning to state *s*′ given action *a* using the state transition probability *Pr*(*s*′|*s*, *a*) which is then weighted by the prior belief probability on state *s* given by *b*(*s*). The resulting sum represents the probability of reaching state *s*′ from all possible states taking into account the prior belief. As stated, the prior belief *b*(*s*) represents our initial belief about the system's state before observing any evidence. It is utilized in the belief update process to weigh the contribution of each possible state to the posterior belief state. States with higher prior probabilities are given more weight in the belief update, reflecting our initial uncertainty about the system's state.

After computing the posterior belief state [30], it is typically normalized to ensure that it remains a valid probability distribution. The resulting belief state from one time step becomes the prior belief state for the next time step, allowing for the propagation of information over time. The definition of the posterior belief defined in Equation (1) can be applied for decision-making cases when there exists infinite amount of time (i.e., infinite horizon). When the decision needs to be made over time steps with finite maximum amount of time *T* (i.e., finite horizon [18] which would be typical for the case of real-time monitoring), the belief update equation for each time step *t* can be written as (*b*_*t*+1_*s*′). At each time step, decision-making actions are taken based on the current belief state, observations, and transition probabilities. The goal is to optimize a specific objective function over the finite horizon, such as maximizing cumulative rewards or minimizing expected costs.

As a demonstration example, let us break down an example for the computational procedure by considering a case of a person whose state *s* can occupy three positions {*A*, *B*, *C*} where the position can be observed by two sensors (*O*_1_, *O*_2_). Let us assume that the initial belief state is defined as a uniform distribution over all the state: *b*(*s*) = {1/3, 1/3, 1/3}. Let us also assume that the person may *move to the right* (*M*_*r*_). Let the state transition models for this action be defined using the following probability distributions: *Pr*(*A*|*M*_*r*_, *A*) = 0.2, *Pr*(*A*|*M*_*r*_, *B*) = 0.7, *Pr*(*A*|*M*_*r*_, *C*) = 0.1, *Pr*(*B*|*M*_*r*_, *A*) = 0.1, *Pr*(*B*|*M*_*r*_, *B*) = 0.6, *Pr*(*B*|*M*_*r*_, *C*) = 0.3, *Pr*(*C*|*M*_*r*_, *A*) = 0.3, *Pr*(*C*|*M*_*r*_, *B*) = 0.4, *Pr*(*C*|*M*_*r*_, *C*) = 0.3. Here, for example, *Pr*(*A*|*M*_*r*_, *B*) = 0.7 implies the conditional probability of the person transitioning to a new position *A* given the person has taken an action *move to the right* who was initially observed at position *B*. The belief state can be updated by simulating the transition models for the chosen action *move to the right* (*M*_*r*_):

 $$\begin{array}{c} Pr\left(b^{\prime }\left|M_{r},b\right.\right)=\left[Pr\left(A\left|M_{r},A\right.\right)\cdot Pr\left(b\left[0\right]\right)+Pr\left(A\left|M_{r},B\right.\right)\cdot Pr\left(b\left[1\right]\right)+Pr\left(A\left|M_{r},C\right.\right)\cdot Pr\left(b\left[2\right]\right),Pr\left(B\left|M_{r},A\right.\right)\cdot Pr\left(b\left[0\right]\right)+Pr\left(B\left|M_{r},B\right.\right)\cdot Pr\left(b\left[1\right]\right)+Pr\left(B\left|M_{r},C\right.\right)\cdot Pr\left(b\left[2\right]\right),Pr\left(C\left|M_{r},A\right.\right)\cdot Pr\left(b\left[0\right]\right)+Pr\left(C\left|M_{r},B\right.\right)\cdot Pr\left(b\left[1\right]\right)+Pr\left(C\left|M_{r},C\right.\right)\cdot Pr\left(b\left[2\right]\right)\right], \end{array}$$

where, by substituting the values defined above, we get the updated belief function as *Pr*(*b*′|*M*_*r*_, *b*) = [0.2 · (1/3) + 0.7 · (1/3) + 0.1 · (1/3), 0.1 · (1/3) + 0.6 · (1/3) + 0.3 · (1/3), 0.3 · (1/3) + 0.4 · (1/3) + 0.3 · (1/3)] = [0.333,0.333,0.333]. After the person transitioned to a new position, the monitoring system receives an observation *O*_1_. The observation model specifies the following probabilities for the observations given the true state as *Pr*(*O*_1_|*A*) = 0.6, *P*(*O*_1_|*B*) = 0.3, *Pr*(*O*_1_|*C*) = 0.1. We now update the belief state using the joint observation model as *Pr*(*b*′|*O*_1_, *M*_*r*_, *b*) ∝ [*Pr*(*O*_1_|*A*) · *Pr*(*b*′[0]), *Pr*(*O*_1_|*B*) · *Pr*(*b*′[1]), *Pr*(*O*_1_|*C*) · *Pr*(*b*′[2])] which we get *Pr*(*b*′|*O*_1_, *M*_*r*_, *b*) ∝ [0.6 · 0.333,0.3 · 0.333,0.1 · 0.333] = [0.2,0.1,0.033]. Normalizing this distribution, we get *Pr*(*b*′|*O*_1_, *M*_*r*_, *b*) ≈ [0.645,0.322,0.033]. This updated belief state reflects the monitoring system's updated knowledge about person's position after taking the *move to the right* (*M*_*r*_) action and receiving the observation *O*_1_. The belief state is used as the basis for subsequent action selection, for example, in the POMDP framework.

#### 3.1.1. Policy

A policy *π*(*a*|*b*) is a mapping from belief state to actions [31]. For example, for *π*(*a*|*b*) = *Pr*(*A*_*t*_ = *a*|*B*_*t*_ = *b*) where *A*_*t*_ is the random variable representing the action taken at time *t*, *B*_*t*_ is the random variable representing the belief state at time *t* and *Pr*(*A*_*t*_ = *a*|*B*_*t*_ = *b*) represents the probability of taking action *a* given the belief state *b*. It specifies what action to take at each belief state, considering the uncertainty about the true state of the system due to partial observability. The policy can be deterministic, where it selects a single action with probability 1 for each belief state, or stochastic, where it assigns probabilities to multiple actions for each belief state [32, 33]. The objective of a policy is to maximize the expected cumulative reward or achieve a specific goal over time under uncertainty. A good policy should make decisions that balance exploration (gathering information about the environment) and exploitation (maximizing rewards based on current knowledge). Policies can consider the entire history of observations and actions to determine the current action. They can also be memoryless and modeled to consider only the current belief state to choose the action. They are simpler but may not always make optimal decisions. In a finite horizon setting, policies are defined for a specific number of time steps or stages. The policy specifies actions to take at each time step to achieve the desired objective over the finite horizon [34].

For example, to define a policy in a POMDP, one needs to specify the action to be taken at each belief state. This can be done using various techniques, such as dynamic programming [35], reinforcement learning [36], or Monte Carlo methods [37]. Dynamic programming methods, like value iteration or policy iteration, involve iteratively updating the value function or the policy until convergence to an optimal solution. Reinforcement learning algorithms, such as Q-learning or deep Q-networks, learn the optimal policy through interaction with the environment, often using techniques like exploration–exploitation strategies and function approximation. Monte Carlo methods, such as Monte Carlo tree search (MCTS), simulate trajectories from the belief state to estimate the value of actions and update the policy accordingly.

Once a policy is defined, it needs to be evaluated to assess its performance. This can be done by simulating the policy in the environment or using techniques like policy evaluation, where the value of each belief state–action pair is computed, and policy improvement, where the policy is updated based on the computed values. Policies in POMDPs are not static; they can be continuously improved over time as more data is collected and better decision-making strategies are learned. Techniques like on-line learning and adaptive strategies allow policies to adapt to changing environments and uncertainties.

Let us again consider a simple example of guiding a person to a destination while facing its partial observability. A policy would dictate the actions through the ambient environment based on the belief about person's position and the destination. If belief state indicates a high probability of the person being near an obstacle, the chosen action by intervening through ambient environment should be such that it guides the person away from obstacles. If the belief state indicates a high probability of being close to the goal, it should choose actions that further encourages move toward the goal. If the belief state indicates uncertainty about the person's location, it may choose actions for exploratory guidance to gather more information. The policy's performance can be evaluated by simulating the movements of the person by success rate of reaching the goal, the time taken, and the number of instances the person faces with obstacles which can be used to assess the effectiveness of the policy. Using the numerical example used in the previous section, the belief state might be represented as a vector [0.3,0.3,0.4], indicating that the decision-maker believes there is a 30% chance of the person being in state *A*, a 30% chance of being in state *B*, and a 40% chance of being in state *C*. Given a belief state, the policy specifies the probability distribution over actions. For example, let us define the policy as follows: *Pr*(*M*_*r*_|*b*) = [0.9,0.9,0.1] (i.e., probability of moving to the right in states *A*, *B*, and *C*, respectively) and *Pr*(*M*_*l*_|*b*) = [0.1,0.1,0.9] (i.e., probability of moving to the left in states *A*, *B*, and *C*, respectively). These probabilities indicate the likelihood of taking each action given the agent's belief about its current state. When the decision-making agent has a belief state, it uses the conditional probability defined by the policy to select an action. For example, if the belief state is [0.3,0.3,0.4], the decision-making agent would calculate the expected utility of each action (e.g., move to the right or move to the left) based on the conditional probabilities defined by the policy and select the action with the highest expected utility.

### 3.2. Reward

The reward function, *R*(*s*, *a*), specifies the immediate reward received when taking action *a* in state *s* [18, 20]. The reward model, *R*, defines the probability distribution over rewards obtained by the agent for taking specific actions in specific states. It can also be expressed as *R*(*r*|*s*, *a*) where *r* is the reward obtained. This model quantifies the desirability or utility of different states and actions, providing feedback to guide its decision-making process [38].

Value function *V*_*t*_(*b*) is used to represent the expected cumulative reward from time *t* onwards given the belief state *b* [39]. As an example, a reward function can be defined recursively as the expected sum of rewards over the remaining time horizon in a finite horizon setting as

(2) $$\begin{array}{c} V_{t}\left(b\right)=E\left[\overset{T-1}{\underset{\tau =t}{\sum }}\gamma^{\tau -t}R\left(s_{\tau },a_{\tau }\right)\left|B_{t}\right.=b\right], \end{array}$$

where *T* is the horizon, *γ* is the discount factor, *s*_*τ*_ is the true state at time *τ*, and *a*_*τ*_ is the action taken at time *τ*.

Decision-making algorithms such as POMDP often involve trade-offs between short-term gains and long-term rewards. Future rewards are typically valued less than immediate rewards due to factors such as uncertainty, risk, and the opportunity cost of delaying gratification. The discount factor 0 < *γ* < 1 defined in Equation (2) serves as a parameter to discount future rewards in decision-making processes. It reflects the preference of the decision-maker for immediate rewards over future rewards and captures the concept of time preference. By discounting future rewards, decision-makers can balance short-term and long-term objectives in their decision-making process. A higher discount factor places more weight on immediate rewards, leading to more myopic decision-making, while a lower discount factor encourages more far-sighted planning. A higher discount factor values immediate rewards more, leading to more impulsive decision-making and shorter planning horizons. A lower discount factor values future rewards more, encouraging longer-term planning and consideration of future consequences.

An optimal policy, denoted as *π*^⋆^(*b*), is a policy that maximizes the expected cumulative reward over the finite time horizon given the belief state *b* [35] or

(3) $$\begin{array}{c} \pi^{⋆}\left(b\right)=\text{argma}{\text{x}}_{\pi }V_{1}\left(b\right), \end{array}$$

where *V*_1_(*b*) is the value function at time *t* = 1, representing the expected cumulative reward from the current time step to the end of the horizon.

One common approach to finding an optimal policy in a finite horizon POMDP is to use the value iteration algorithm. The algorithm iteratively computes the value function for each belief state *b* until convergence [18]. At each iteration, the value function is updated using the Bellman backup equation (Appendix A) [36], which expresses the value of a belief state in terms of the values of its successor states:

(4) $$\begin{array}{c} V_{t}\left(b\right)=\text{ma}{\text{x}}_{a}\left(\underset{s^{\prime }}{\sum }T\left(s^{\prime }\left|b,a\right.\right)\cdot \left(R\left(s,a\right)+\gamma \cdot V_{t+1}\left(b^{\prime }\right)\right)\right). \end{array}$$

The algorithm terminates when the change in the value function between iterations falls below a predefined threshold.

Once the value function has converged, the optimal policy can be extracted from it. For each belief state *b*, the optimal action *a*^⋆^ is determined by selecting the action that maximizes the expected cumulative reward according to the value function from Equation (4) or *π*^⋆^(*b*) = argmax_*π*_(*V*_*t*_(*b*)). The resulting policy represents the optimal action to take at each belief state to maximize the expected cumulative reward over the finite time horizon. The value iteration algorithm and policy extraction process can be repeated whenever the environment or task conditions change. This allows for continuous improvement of the policy based on new information or updated reward structures.

Following similar simple numerical example as in the previous section, we are assuming a person state *s* can occupy three positions {*A*, *B*, *C*}, with available ambient actions {*M*_*r*_, *M*_*l*_} and with belief state *b*(*s*) = {0.3,0.3,0.4}. We also define a reward function as assigning rewards for reaching certain states, such as +10 for reaching state *C*, 0 otherwise. Lets us also assume the following transition models: *Pr*(*A*|*M*_*r*_, *A*) = 0.9, *Pr*(*B*|*M*_*r*_, *A*) = 0.1 and observation models as *Pr*(*O*_1_|*A*) = 0.8, *Pr*(*O*_1_|*B*) = 0.2, where the position can be observed by two sensors (*O*_1_, *O*_2_). Let us assume the reward of reaching state goal state *C* with +10 and 0 otherwise. The transition model specifying the following probability distribution with a small chance of failure, for example, *Pr*(*A*|*M*_*r*_, *A*) = 0.9, *Pr*(*B*|*M*_*r*_, *A*) = 0.1. With the observation model, *Pr*(*O*_1_|*A*) = 0.8, *Pr*(*O*_1_|*B*) = 0.2. The person can transition to states *A*, *B*, or *C* with certain probabilities given the ambient cue action *M*_*r*_. For each possible transition, we calculate the expected utility as the product of the probability of the transition, the probability of receiving the reward in the resulting state, and the reward itself. We sum up these expected utilities for all possible transitions to get the overall expected utility for the ambient cue action *M*_*r*_. Similarly, we calculate the expected utility for the ambient cue action *M*_*l*_ by considering all possible transitions and their associated rewards. For the action *M*_*r*_, expected utility = (0.9 · 0.3 · 0) + (0.1 · 0.3 · 0) + (0.1 · 0.4 · 0) + (0.9 · 0.4 · 10) = 3.6. For the action *M*_*l*_, expected utility = (0.1 · 0.3 · 0) + (0.9 · 0.3 · 0) + (0.9 · 0.4 · 0) + (0.1 · 0.4 · 10) = 0.4. Since the action *M*_*r*_ has a higher expected utility (3.6) compared to (0.4), the guidance system would select *M*_*r*_ as the guiding action for the person resulting in the highest expected utility given the belief state [0.3,0.3,0.4].

### 3.3. Belief Tree

In the context of POMDP implementation and solution, a belief tree [20] is a data structure used as a part of the sampling-based approximation to represent and efficiently explore the space of possible belief states in a partially observable environment [40, 41]. It commonly employed various greedy algorithms for solving POMDPs, such as the MCTS algorithm. In the following, we define some of the common terminologies which are used when referring to the belief tree. *Nodes:* Each node in the belief tree represents a specific belief state, for example, associated with the monitoring person. As stated, a belief state is a probability distribution over the set of possible states, indicating the likelihood of the person being in each state given its observations and previous actions. The root node of the tree typically represents the initial belief state, while the leaf nodes represent terminal belief states. *Edges:* Edges in the belief tree represent actions taken to transition between belief states. The edge may also contain additional information, such as transition probabilities and immediate rewards associated with action taking. *Exploration:* The belief tree is constructed and expanded through a process of exploration, where all possible actions are considered and their resulting belief state transitions. During exploration, potential action sequences from the current belief state are simulated, updating the belief tree with new nodes and edges as it explores different branches of the tree. *Pruning:* To manage computational complexity and focus exploration on promising branches, belief trees are often pruned. Pruning involves selectively discarding or collapsing branches of the tree that are unlikely to lead to optimal solutions. This pruning process helps to concentrate computational resources on regions of the belief space that are more likely to yield high-quality solutions. *Search and optimization:* Once the belief tree is constructed, search algorithms, such as MCTS, are used to traverse the tree and identify promising action sequences. These algorithms, for example, [39, 40], are aimed at finding the optimal policy or action sequence that maximizes the agent's expected cumulative reward over time. By efficiently exploring and evaluating different branches of the belief tree, these algorithms can effectively address the challenges of planning and decision-making in partially observable environments.

**Figure 1:**
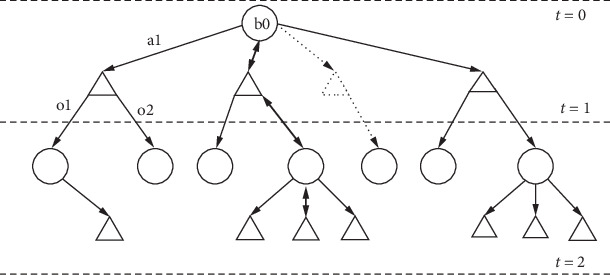
An example of belief tree construction for sampling-based on-line algorithm. Algorithms such as MCTS iteratively build the search tree. Starting with the initial belief in the selection phase, it traverses the tree and selects a child node that maximizes a policy. The algorithm continues until it reaches a leaf node or a terminal node when the algorithm backpropagates by updating the belief tree statistics.

A typical implementation of MCTS algorithm follows a sequence of steps in order to explore and search the belief tree to find an optimal policy (Figure 1). (a) *Initialization*: It first defines a root node representing the initial belief state. *Selection*: it then traverses the tree from the root node downward using a selection policy (such as upper confidence bound (UCB)) [41]. At each step, select the child node that maximizes a selection criterion balancing between exploration and exploitation. This criterion typically considers both the value of the node and the number of visits. *Expansion:* If the selected node has not been fully expanded (i.e., not all possible actions or observations have corresponding child nodes), the algorithm expands it by adding one or more child nodes representing possible actions or observations. If the node has been fully expanded, proceed directly to the next step which is *simulation (rollout)* step where once a leaf node is reached (either expanded or fully expanded), perform a simulation (rollout) from that node. Simulate actions and observations until reaching a terminal state or a predefined depth. The rollout typically uses a heuristic or random policy to estimate the value of the node and finally the *backpropagation* step [38] where the algorithm updates the statistics of all nodes visited during selection and expansion. Propagate the outcome of the simulation (e.g., reward or value estimate) back up the tree. Increment the visit count of each node traversed during selection. Update the value estimate of each node based on the outcome of the simulation. The algorithm then repeats the selection, expansion, simulation, and backpropagation steps for a predefined number of iterations or until a termination condition is met.

## 4. Drunkard's Walk

In this section, we explore and study the drunkard's walk [42] as an example where its model is based on the notion of MC and its extensions to hidden MC can be used as a decision-making framework for ambient guidance. A drunkard's walk, or a random walk, is a stochastic process where the walker moves randomly from one state to another. In this example, we propose a MC that describes a sequence of events where the probability of transitioning to any particular state depends solely on the current state and not on the sequence of events that preceded it. To model a drunkard's walk as a MC, a set of states representing different positions of the drunkard can be defined. The transitions between these states would be determined by a set of probabilities associated with each state. In the context of a drunkard's walk, the probabilities would represent the likelihood of moving to a neighboring position. Here, for example, in a grid planar world, states can be its different positions. Transition probabilities assign probabilities for moving from one state to another where a collection of these transition can be captured in a transition matrix where each entry (*i*, *j*) corresponds to the probability of transitioning from state *i* to state *j* where, at each step, the system transitions from one state to another based on the probabilities in the transition matrix.

**Figure 2:**
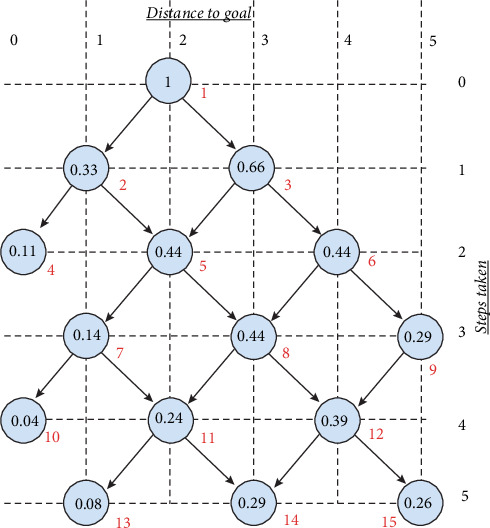
A sample state sequence of drunkard's walk where the initial position is defined at Position 2 (*s*_2_) in a hallway with a fixed prior transition probability distribution of move to the right with probability of 2/3 and move to the left with probability of 1/3. The horizontal grid shows the interval distances (depth) of the graph to the discrete goal locations and vertical grid shows the step sequences. The number in each node indicates the joint probability of reaching to the particular goal location at a given step in the sequence.

Figure 2 shows an example graph diagram of a 1D drunkard's walk compromising 5 discrete states (*i* = 1, *n* where *n* = 5) which can be 5 locations along a hallway of a living space which also has 5 instances of observations at these states (*t*_*i*_ where *i* = 1–5). The graph of this example also depicts a case where the walk initialized at location when *i* = 2. A physical example would be a person entering in a long hallway with five observation states at state and observation *i* = 2. In this example, we also assume that the person has a continuous tendency (probability) to move to the right with *Pr*_*r*_ = 2/3 and to the left with *Pr*_*l*_ = 1/3 where *Pr*_*r*_ + *Pr*_*l*_ = 1. Given this description and further assuming that each step in the walk is memoryless and independent, one can deduce from the MC description, and for example starting from the initial state, the probability of the person being at some observation point (goal point) after a number of steps taken using the following Markov relationship: *Pr*(*s*_1_, *s*_2_, ⋯, *s*_*n*_) = *Pr*(*s*_1_)∏_*i*=2_^*i*=*n*^*Pr*(*s*_*i*_|*s*_*i*−1_). For example, starting from the initial state when *i* = 2 and with *Pr* = 1 (Node 1 in Graph 2), we can compute the probability of the person reaching the goal state *i* = 0 (i.e., Node 10 of the graph of Figure 2) after four instances of observation. It can be seen from the graph that the state *i* = 0 (Node 10) can be reached following two distinct paths from the starting state; these are *s*_2_⟶*s*_1_⟶*s*_0_ (or using node numbering: 1⟶2⟶4) and *s*_2_⟶*s*_3_⟶*s*_2_⟶*s*_1_⟶*s*_0_ (or using node numbering: 1⟶3⟶5⟶7⟶10). Using properties of MC, we can compute the probability of reaching goal state of node 10 after four instances as

(5) $$\begin{array}{c} \left(Pr\left(s_{2}⟶s_{1}⟶s_{0}\right)=Pr\left(s_{2}\right)Pr\left(s_{1}\left|s_{2}\right.\right)Pr\left(s_{0}\left|s_{1}\right.\right)\right)+ \end{array}$$

(6) $$\begin{array}{c} \left(Pr\left(s_{2}⟶s_{3}⟶s_{2}⟶s_{1}⟶s_{0}\right)=Pr\left(s_{3}\left|s_{4}\right.\right)Pr\left(s_{2}\left|s_{3}\right.\right)Pr\left(s_{1}\left|s_{2}\right.\right)Pr\left(s_{0}\left|s_{1}\right.\right)\right)=\frac{1}{9}+\frac{4}{81}=16\%, \end{array}$$

which implies that given the tendencies of the drunkard in moving to the right with *Pr*_*r*_ = 2/3 and *Pr*_*l*_ = 1/3, there exists 16% chance of the walk reaching Node 10 of the graph (or Station 0 in the long hallway). Similar computation can be carried in reaching state *s*_5_ after five instances (*t*_5_) to 8/27 + 64/243 = 55% (i.e., probability of Node 9 plus probability of Node 15). This implies that given the initial movement tendencies and its initial position of the person, there exists a high probability of reaching state *s*_5_ after five steps than reaching state *s*_0_ after four steps.

**Figure 3:**
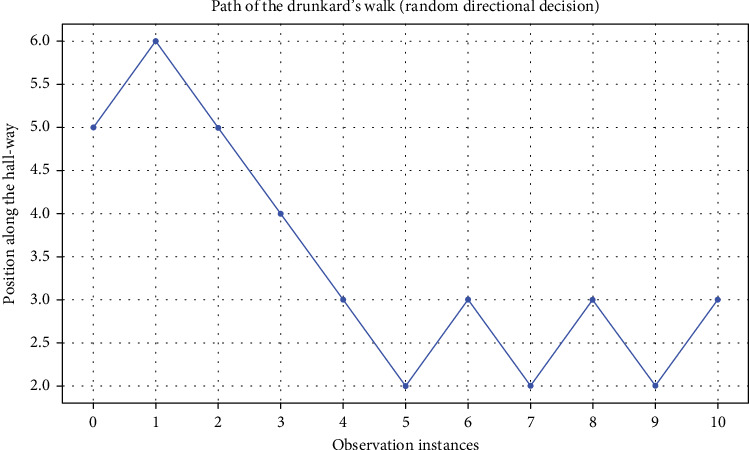
An example trajectory of drunkard's walk starting at Position 5 where the selection between left and right transition is fully random.

**Figure 4:**
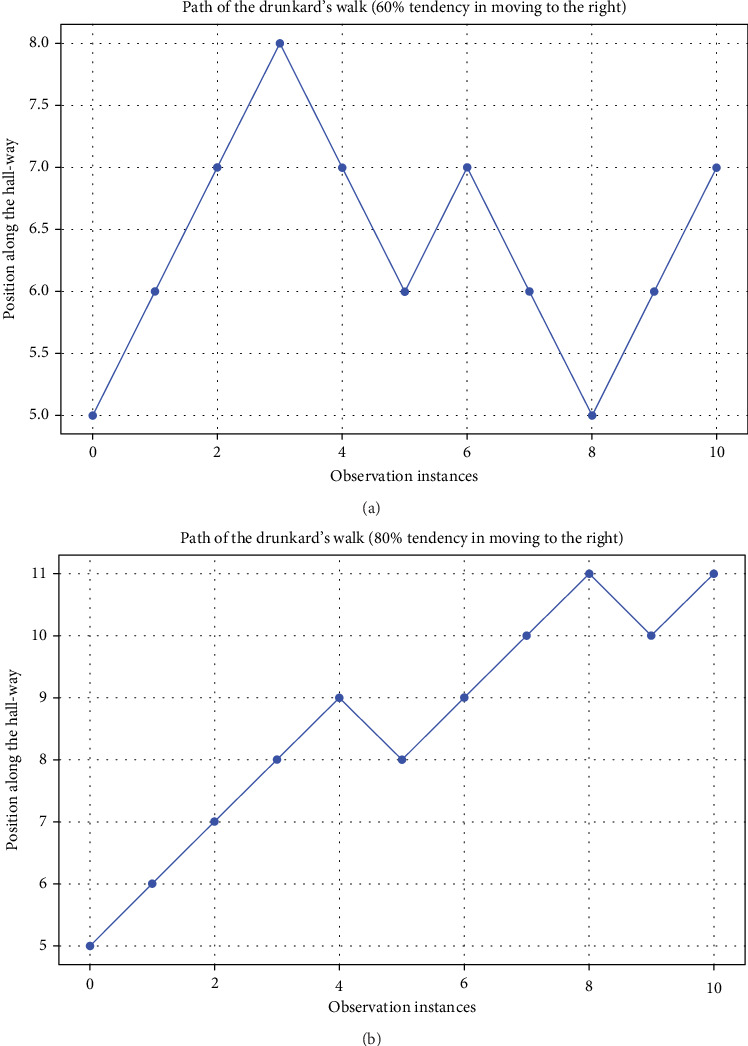
Examples of drunkard's walk starting at Position 5 where the tendency for moving to the right is adapted to 60% in (a) and then to 80% in (b).

Figure 3 shows an example of drunkard's walk where the selection between moving to the right or left is purely random. However, as it can be seen in Figure 4, if it is possible to motivate/encourage/guide the direction of movements so there is a higher tendency and probability for the movement to the right, it is possible to reach, for example, a goal location which is located to the right. This can be seen in Figure 4, where the probability of moving to the right increases from 60% to 80%.

The above open-loop example demonstrates on how the framework of MC can be used to establish a proper model of movements and observing the effects of the prior probability distribution of tendency of movements. A trivial observation of the above is the demonstration of how prior local probability distribution of state transition can have an effect in the global movement patterns of the drunkard's walk.

The basis of the 1D drunkard's walk example can be extended as HMMs where the observable events in the HMM correspond to the movements or positions of the drunkard at any given instances which is captured through assigned emission probabilities to each of the state, representing the likelihood of observing a particular movement or position given the hidden (or latent) state. For a drunkard's walk, these probabilities might reflect the erratic and unpredictable nature of the walk. Here, the observable sequence is the actual sequence of movements or positions observed during the walk. The objective of the ambient guiding system is through environmental cues and/or action of the guiding robot to provide feedback which can affect the drunkard's walk toward a specific goal.

**Figure 5:**
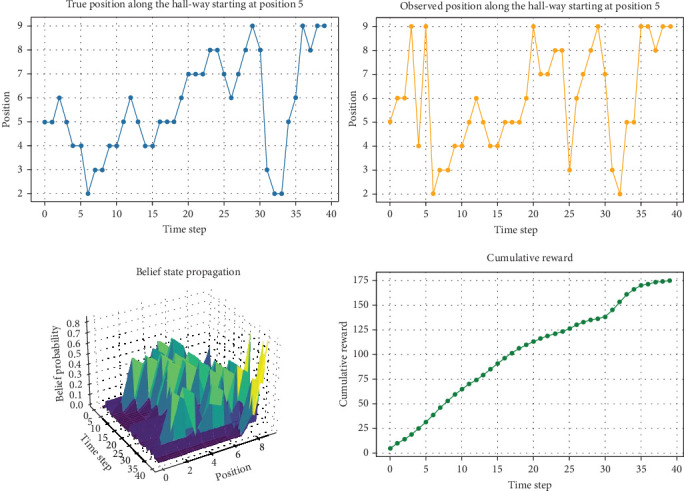
A simple example of POMDP implementation where the walk is initiated at Position 5 with high belief probability. The observed position is contaminated with noise and accumulated reward for the drunkard in selecting a direction toward increased position increment is shown. The 3D plot shows the changing of the belief probability distribution of the position at each time step of simulation.

Figure 5 shows simple simulation results of implementation of POMDP for guiding the drunkard to the right. The 3D plot of probability serves as a visual representation of the belief state over time, where each point on the grid represents the probability of the drunkard's position at a specific time step. This plot captures the uncertainty inherent in the system, showcasing how the belief state evolves as the drunkard moves through the environment and receives noisy observations. The peaks in the probability distribution indicate regions where the drunkard is most likely to be located, while the overall shape of the distribution reflects the level of confidence in these estimations. Additionally, the true and observed position plots offer insights into the effectiveness of the guiding system. Discrepancies between the true and observed positions highlight instances of observation error, emphasizing the importance of robust inference mechanisms for accurate state estimation. This simple study provides an overview of the guiding process, offering valuable insights into the challenges and potential solutions for developing ambient guiding systems for individuals with complex behavioral patterns like a drunkard's walk.

## 5. Discussions and Conclusions

The convergence of ambient intelligence and persuasive technology is revolutionizing the interaction between information technology and cognitive science. This fusion is paving the way for the creation of smart environments that can not only intelligently respond to human actions but also influence behavior. This significant evolution necessitates a re-evaluation of the traditional frameworks used to understand the relationship and role distribution between humans and technological artifacts. By understanding and critically examining both the advantages and potential risks associated with these emerging technologies, it becomes possible to establish more feasible and practical frameworks. Such frameworks are essential for effectively integrating these advanced technologies into daily life while ensuring ethical considerations and user acceptance. Several innovative approaches have been developed that integrate models of human movement and activities with external ambient guiding systems. These approaches often utilize implicit modeling assumptions and methodologies akin to those found in the POHMM framework, such as POMDPs. These models are particularly adept at handling the uncertainty and variability inherent in human behavior and environmental interactions.

Recent advancements in various solutions to POHMM and its extensions can effectively work with generative learning models by incorporating them into the decision-making process to approximate optimal policies. Generative learning models can be utilized to estimate the transition probabilities, observation probabilities, and reward distributions from data or domain knowledge. Techniques such as maximum likelihood estimation, Bayesian inference, or machine learning algorithms like neural networks can be employed to learn these models from historical data or simulations. Generative models can be combined with domain-specific knowledge or expert insights to enhance the performance of solvers. Incorporating prior knowledge about the environment, task structure, or agent preferences into the generative models can lead to more informed decision-making and improved solution quality.

Utilizing sensors like cameras, motion detectors, or IoT devices can provide valuable information about the environment. These sensors can be part of the observation model, enhancing the model's ability to understand the current state. Including context-specific data such as time of day, user preferences, or historical patterns helps refine the model's predictions and recommendations. Extensions may involve adapting the model over time based on learning from user behavior. This adaptability ensures that the guidance system becomes more personalized and accurate as it interacts with the user and the environment. The model can be trained to guide individuals through ambient medium and through their living space, considering factors like optimal paths, avoiding obstacles, and adapting to changes in the environment. By analyzing historical data, the model can predict user behavior, such as preferred routes, common destinations, or times of high activity. For individuals with specific needs, such as the elderly or those with mental mobility challenges, the system can adapt guidance strategies to provide tailored support. Incorporating sensor data raises privacy concerns. It is crucial to design the system with privacy-preserving measures to ensure user trust and compliance with regulations. Efficient algorithms are required for real-time processing, especially in dynamic environments where the living space may undergo rapid changes.

This paper presents a comprehensive overview and description of the POHMM framework such as POMDP, highlighting its key components and expanding on how they can be interpreted in designing and developing movement monitoring and ambient guidance system. The key components of such framework include the following:

• Belief states: The system maintains a probabilistic representation of the current state of the world, given the partial observability of the environment. This allows the system to make informed decisions even with incomplete information about the user's exact state.
• Action selection: The POMDP framework enables the selection of optimal actions based on expected outcomes. By considering both immediate and future rewards, the system can guide users in a way that balances short-term convenience with long-term benefits such as safety and efficiency.
• Observation models: These models describe the likelihood of various observations given the current state and action taken. They are crucial for updating belief states and refining the system's understanding of the user's context [11, 17].
• Reward functions: The framework defines rewards for different state–action pairs, guiding the system to promote desirable behaviors and outcomes. In the context of an ambient guidance system, rewards might be designed to encourage safe, efficient, and user-friendly navigation.
• Policy optimization: POMDPs allow for the optimization of policies that specify the best actions to take in various belief states. This ensures that the system consistently provides high-quality guidance tailored to the user's needs and the environmental context.

By synthesizing these components, such framework offers a robust foundation for designing advanced movement monitoring and ambient guidance systems. These systems can intelligently adapt to users' changing contexts, providing personalized and effective guidance that enhances overall user experience and safety.

In conclusion, the integration of ambient intelligence and persuasive technology through frameworks like POMDP holds immense potential for transforming human–technology interactions. By leveraging the strengths of these models, we can develop sophisticated systems that not only respond to but also anticipate and influence human behavior, leading to smarter, more responsive environments. This paper is aimed at providing a detailed exploration of these frameworks, offering insights into their application and potential to contribute to movement monitoring and ambient guidance systems.

## Data Availability

The data that used to support the findings of this study are available from the corresponding author upon request.

## Appendix A: The Bellman Backup Equation in POMDPs

The Bellman backup equation is a fundamental concept in dynamic programming [43], used to compute the value function of a policy by expressing the value of a state in terms of the values of its successor states. In the context of POMDPs, the Bellman backup equation is used to iteratively update the value function for each belief state, guiding the selection of actions to maximize expected cumulative rewards [20].

In the context of a finite horizon POMDP, the value function *V*_*t*_(*b*) represents the expected cumulative reward from time *t* onwards given a belief state *b*. Value iteration is aimed at iteratively computing the value function for each belief state until convergence. At each iteration, the value function is updated based on the Bellman backup equation. The Bellman backup equation for a belief state *b* at time step *t* is derived by considering the expected cumulative reward obtained by taking an action *a* in the current belief state *b* and observing the resulting successor state *s*′. Consider the expected cumulative reward from time step *t* onwards given belief state *b* and action *a*. It can be expressed as

 $$\begin{array}{c} V_{t}\left(b\right)=E\left[\overset{T-1}{\underset{\tau =t}{\sum }}\gamma^{\tau -t}R\left(s_{\tau },a_{\tau }\right)\left|B_{t}\right.=b\right]. \end{array}$$

Using the law of total expectation, we can express this expectation in terms of the transition probabilities *T*(*s*′|*b*, *a*) and immediate rewards *R*(*s*, *a*) as

 $$\begin{array}{c} V_{t}\left(b\right)=\underset{s^{\prime }}{\sum }T\left(s^{\prime }\left|b,a\right.\right)\left(R\left(s,a\right)+\gamma \cdot E\left[\overset{T-1}{\underset{\tau =t+1}{\sum }}\gamma^{\tau -t-1}R\left(s_{\tau },a_{\tau }\right)\left|B_{t+1}\right.=b^{\prime }\right]\right), \end{array}$$

where *b*′ is the belief state at time *t* + 1 after taking action *a*. Introducing a maximization step over all possible action *a* to select the action that yields the highest expected cumulative reward for the current belief state

 $$\begin{array}{c} V_{t}\left(b\right)=\text{ma}{\text{x}}_{a}\left(\underset{s^{\prime }}{\sum }T\left(s^{\prime }\left|b,a\right.\right)\left(R\left(s,a\right)+\gamma \cdot V_{t+1}\left(b^{\prime }\right)\right)\right). \end{array}$$

This step yields the Bellman backup equation, which expresses the value of a belief state *b* at time step *t* as the maximum overall possible actions. The optimal policy can be selected by choosing the action that maximizes the expression inside the maximum operator for each belief state. This results in the optimal policy that maximizes the expected cumulative reward over the finite time horizon.
